# Fire facilitates ground layer plant diversity in a Miombo ecosystem

**DOI:** 10.1093/aob/mcae035

**Published:** 2024-03-04

**Authors:** Jakub D Wieczorkowski, Caroline E R Lehmann, Sally Archibald, Sarah Banda, David J Goyder, Mokwani Kaluwe, Kondwani Kapinga, Isabel Larridon, Aluoneswi C Mashau, Elina Phiri, Stephen Syampungani

**Affiliations:** School of GeoSciences, The University of Edinburgh, Edinburgh EH8 9XP, UK; Tropical Diversity, Royal Botanic Garden Edinburgh, Edinburgh EH3 5LR, UK; School of GeoSciences, The University of Edinburgh, Edinburgh EH8 9XP, UK; Tropical Diversity, Royal Botanic Garden Edinburgh, Edinburgh EH3 5LR, UK; Centre for African Ecology, School of Animal, Plant and Environmental Sciences, University of the Witwatersrand, Johannesburg 2050, South Africa; Centre for African Ecology, School of Animal, Plant and Environmental Sciences, University of the Witwatersrand, Johannesburg 2050, South Africa; Herbarium, Division of Forest Research, Forestry Department, PO Box 22099, Kitwe, Zambia; Royal Botanic Gardens, Kew, Richmond, Surrey TW9 3AE, UK; Herbarium, Division of Forest Research, Forestry Department, PO Box 22099, Kitwe, Zambia; Dag Hammarskjöld Institute for Peace and Conflict Studies – Environment, Sustainable Development and Peace, Copperbelt University, PO Box 21692, Kitwe, Zambia; Royal Botanic Gardens, Kew, Richmond, Surrey TW9 3AE, UK; Centre for African Ecology, School of Animal, Plant and Environmental Sciences, University of the Witwatersrand, Johannesburg 2050, South Africa; Foundational Research and Services, South African National Biodiversity Institute (SANBI), Private Bag X101, Pretoria 0184, South Africa; Herbarium, Division of Forest Research, Forestry Department, PO Box 22099, Kitwe, Zambia; Oliver R Tambo Africa Research Chair Initiative for Environment and Development, Copperbelt University, PO Box 21692, Kitwe, Zambia; Department of Plant and Soil Sciences, University of Pretoria, Private Bag X20, Hatfield, Pretoria 0028, South Africa

**Keywords:** Miombo, fire regime, biodiversity, encroachment, ground layer, herbaceous, plant functional group, species richness, savanna, fire management, C_4_ grass, geoxyle

## Abstract

**Background and Aims:**

Little is known about the response of ground layer plant communities to fire in Miombo ecosystems, which is a global blind spot of ecological understanding. We aimed: (1) to assess the impact of three experimentally imposed fire treatments on ground layer species composition and compare it with patterns observed for trees; and (2) to analyse the effect of fire treatments on species richness to assess how responses differ among plant functional groups.

**Methods:**

At a 60-year-long fire experiment in Zambia, we quantified the richness and diversity of ground layer plants in terms of taxa and functional groups across three experimental fire treatments of late dry-season fire, early dry-season fire and fire exclusion. Data were collected in five repeat surveys from the onset of the wet season to the early dry season.

**Key Results:**

Of the 140 ground layer species recorded across the three treatments, fire-maintained treatments contributed most of the richness and diversity, with the least number of unique species found in the no-fire treatment. The early-fire treatment was more similar in composition to the no-fire treatment than to the late-fire treatment. C_4_ grass and geoxyle richness were highest in the late-fire treatment, and there were no shared sedge species between the late-fire and other treatments. At a plot level, the average richness in the late-fire treatment was twice that of the fire exclusion treatment.

**Conclusions:**

Heterogeneity in fire seasonality and intensity supports diversity of a unique flora by providing a diversity of local environments. African ecosystems face rapid expansion of land- and fire-management schemes for carbon offsetting and sequestration. We demonstrate that analyses of the impacts of such schemes predicated on the tree flora alone are highly likely to underestimate impacts on biodiversity. A research priority must be a new understanding of the Miombo ground layer flora integrated into policy and land management.

## INTRODUCTION

Savanna ecosystems worldwide are changing rapidly through land-use change and intensification, shifting fire regimes, encroachment and climate change (Phelps *et al.*, 2022; Stevens *et al.*, 2022). Via their unique biodiversity, savanna ecosystems deliver numerous ecosystem services supporting the livelihoods of millions of people (Ryan *et al.*, 2016). Ground layer plant diversity is an important but understudied component of savanna ecosystems, making important contributions to ecosystem functioning and provisioning and regulating ecosystem services via pollination, materials, foods and medicines (Stevens *et al.*, 2022). More often than not, however, analyses of savanna plant community richness, composition and turnover tend to focus on woody plants (e.g. Kikula, 1986; Chidumayo, 1997; Amoako *et al.*, 2023). A consequence of analyses prioritizing one component of a flora is potential misinterpretation of ecosystem dynamics, whereby plant taxa and functional groups that comprise ecosystems have different or divergent responses to changes in the environment, such as fire regimes. Therefore, a holistic understanding of floral diversity, inclusive of ground layer plants, is needed to understand ecosystem dynamics and responses to fire.

Fire shapes many savanna ecosystems worldwide, whereby it promotes landscape heterogeneity in woody cover and whereby recurrent fire maintains open canopies and a grass-dominated ground layer (Bond *et al.*, 2005; Sankaran *et al.*, 2008; Ryan and Williams, 2011; Lehmann *et al.*, 2014). In fire-adapted savanna, fire exclusion can lead to an increase in woody biomass and cover and the loss of C_4_ grass-dominated ground layers, associated with diminished ground layer light availability and changing microclimates (Hoffmann *et al.*, 2012; Pilon *et al.*, 2021). Analyses of floral diversity in response to fire regimes have been conducted particularly across savannas of North and South America (e.g. Bowles and Jones, 2013; Pinheiro *et al.*, 2016; Abreu *et al.*, 2017; Brewer and Zee, 2021), usually pointing to a decrease in herbaceous species richness under fire exclusion when followed by woody encroachment (Wieczorkowski and Lehmann, 2022). However, the focus is often on trees and grasses, and less to little is known about the responses of other functional groups within savannas and where different groups are not expected to respond uniformly to pyrodiversity (He *et al.*, 2019). The C_4_ grasses are the functionally dominant element in the savanna ground layer, abundant in fire-intensive environments, characterized by fire–grass positive feedbacks (D’Antonio and Vitousek, 1992), with consistent evidence of a decline in C_4_ grass richness under fire exclusion (Peterson and Reich, 2008; Bowles and Jones, 2013; Diaz-Toribio *et al.*, 2020). The C_3_ grasses are uncommon in tropical savannas but can be common in shaded sites and in sites of fire exclusion reflecting a transition from open savanna to closed forest (Ratnam *et al.*, 2011). Nevertheless, C_3_ grass species richness might still increase with high fire frequency in some environments (e.g. Bowles and Jones, 2013). Savanna forbs, which include dicot and monocot species, are usually suited to frequent fire disturbance (Uys, 2006; Siebert and Dreber, 2019), e.g. through post-fire flowering (Bond, 2016). Long-term fire exclusion has been documented to be correlated with declines in forb richness (Woinarski *et al.*, 2004; Peterson and Reich, 2008). Other functional groups, such as sedges, geoxyles and ferns, also have fire adaptations (Kornaś, 1978; Medwecka-Kornaś and Kornaś, 1985; Maurin *et al.*, 2014), but data on the impact of fire manipulation on local richness patterns are limited. Geoxyles are plants that resprout after disturbance from buds located on substantive long-lived belowground structures (Pausas *et al.*, 2018), and many are well adapted to fire (Maurin *et al.*, 2014). Ferns have a diversity of strategies (flammable fire dependent, fire tolerant or fire sensitive), enabling the colonization of diverse open and closed environments (Mehltreter *et al.*, 2010); therefore, their responses can vary depending on the suite of traits characterizing local species. Although the global evidence on the impact of different fire regimes on ground layer plant diversity allows for generalized predictions, there are limitations to the conclusions that can be drawn, because savannas of different regions differ in their floral diversity and fire regimes (Lehmann *et al.*, 2014) and there are no data for the Miombo region of Africa.

Within African savannas, fire management is a hot topic related to the development of carbon offsetting and sequestration schemes involving fire exclusion, fire management or policies for planting trees (e.g. Veldman *et al.*, 2019; Russell-Smith *et al.*, 2021; Tear *et al.*, 2021). Fire suppression is impractical, because long-term litter accumulation combined with prolonged dry seasons facilitate accidental high-intensity fires that significantly reduce woody biomass (Chidumayo, 1997). Early dry-season fires are often advocated, because at times of higher fuel moisture contents and relatively mild weather, fires are of low intensity and thereby tend to be small in size and, consequently, relatively easy to manage (Govender *et al.*, 2006; Laris and Wardell, 2006). Early dry-season fires have also been suggested to support a more spatially pyrodiverse landscape in comparison to late dry-season fires (Laris, 2005), burning non-homogeneously and creating patches within the landscape (Parr and Andersen, 2006). In contrast, late dry-season fires are more intense and usually larger, significantly reducing the woody biomass and opening the canopy (Govender *et al.*, 2006; Oliveira *et al.*, 2015; Archibald, 2016). However, widespread application of uniform early dry-season fire is unlikely to control woody encroachment (Smit *et al.*, 2016), especially in a high-CO_2_ world (Stevens *et al.*, 2017), and might not be beneficial to all components of the biodiversity (Chambers and Samways, 1998; Kone *et al.*, 2018). Seasonal, mosaic burning of vegetation, with a combination of early, late and no burning, is common in some landscapes, but the understanding of its effects on biodiversity is limited (Laris, 2011). The potential of pyrodiversity to support plant diversity is currently suggested to be largely context specific (Gordijn and O’Connor, 2021). Therefore, regional experiments on contrasting fire regimes provide valuable insights into the effects of fire on plant diversity and consequent changes to ecosystem structure to inform fire management.

The Miombo ecoregion of Africa is an epicentre of land-use transformation and an expanding agricultural frontier (Ryan *et al.*, 2016; Osborne *et al.*, 2018), but there is still much to understand about the ecology of the region, urgently needed for evidence-based land policy and management. The Miombo, spanning ~3 × 10^6^ km^2^, is often described as the largest savanna region in the world (Campbell *et al.*, 1996), although it is variably also interpreted as a dry forest (e.g. Frost, 1996; Pelletier *et al.*, 2018; Santoro *et al.*, 2021; Rozendaal *et al.*, 2022). Fire shapes Miombo ecosystem dynamics and has been a characteristic and integral feature of the region for at least the last 200 000 years (Lawton, 1978). In Miombo ecosystems, frequent fire facilitates canopy openness (Furley *et al.*, 2008), and fire suppression results in closed-canopy formations (Trapnell, 1959; Chidumayo, 1988). The richness of both woody and herbaceous species across the Miombo, and especially in Zambia, points to a region of diversity and endemism (Ribeiro *et al.*, 2020; Vollesen and Merrett, 2020). However, no in-depth ground layer plant diversity analyses of Miombo vegetation have been published to date, although they are required to understand how fire shapes floristic diversity across the broad range of taxa that compose these ecosystems. It is unclear whether patterns in Miombo align or diverge from those observed in other savannas.

Here, we quantify ground layer plant diversity in a Miombo ecosystem in response to 60 years of fire manipulation comprising exclusion (no-fire treatment), annual early dry-season fire (early-fire treatment) and annual late dry-season fire (late-fire treatment). The fire experiment in Mwekera, Zambia, along with other such experiments in Africa established by forestry departments, tend to monitor trees rather than herbaceous plants. Although these ecosystems are recognized as potentially having diverse ground layers, data on ground layer plant richness and composition are scarce to non-existent, with herbaceous communities not previously assessed quantitatively, other than by mentions of individual species (e.g. Frost, 1996; Shelukindo *et al.*, 2014). Driven by these knowledge gaps, we address two questions. First, how does fire alter plant species composition in Miombo ecosystems? We assess β-diversity across the three fire treatments and also distinguish patterns among seven groups: C_4_ grasses, C_3_ grasses, sedges, non-graminoid monocots, dicots, ferns and geoxyles. We then compare patterns of ground layer composition with trees. Second, what is the impact of fire on species richness, and are these impacts uniform across plant functional groups? We assess richness responses using species richness data from 21 1-m-diameter plots set up in each fire treatment. We use the seven groups listed, because little is known of the ecology of the flora, and we expect disparity in fire responses and thus important differences masked by analyses of total species richness.

## MATERIALS AND METHODS

### Study site and experimental design

**Fig. 1.**
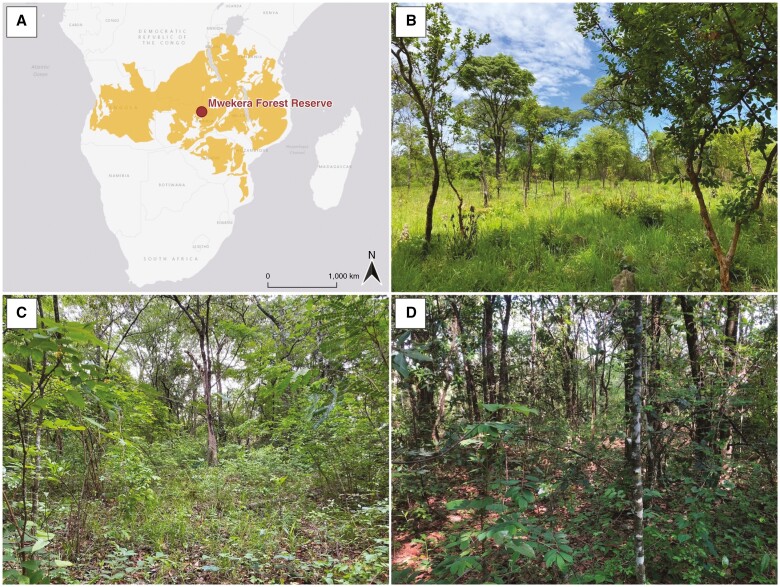
(A) Location of the study site of Mwekera Forest Reserve in the Copperbelt region, Zambia (12.842°S, 28.366°E) within the Miombo region (highlighted area; Miombo extent was delineated based on Olson *et al.*, 2001). (B–D) Photographs of three experimental treatments in the studied Miombo ecosystem in December 2022: late-fire (B), early-fire (C) and no-fire (D). Photograph credits: A. P. Courtenay and C. E. R. Lehmann.

Mwekera Forest Reserve No. 6 in the Copperbelt region of Zambia (Fig. 1A) is situated at an elevation of 1220 m a.s.l., with mean annual rainfall of 1228 mm and a dry season from April to November and rainy season from December to March (Kalaba *et al.*, 2013; Fick and Hijmans, 2017). The mean minimum and maximum daily temperatures in the coolest period (May–July) are 7 and 25 °C, respectively, and in the warmest month (October) they are 21 and 32 °C, respectively (Chidumayo, 1997).

The Mwekera fire experiment was established in 1960 by Forest Research. An old-growth Miombo ecosystem was clear felled but not ploughed, keeping the ground layer intact (Chidumayo, 1997). We will refer to the studied ecosystem as the ‘Miombo ecosystem’ instead of the common ‘Miombo woodland’ to encompass the diversity of vegetation structures resulting from different fire-management strategies, spanning from open savanna to closed dry forest. The fire experiment was established for training and research in fire management to determine the response of a Miombo ecosystem to different fire regimes. The experimental site was chosen for its homogeneity as a wet Miombo ecosystem (mean annual rainfall > 1000 mm according to White, 1983), on relatively level ground without a distinct aspect and with good drainage conditions. The soils are sandy, deep and nutrient poor, typical of plateau soils in the Copperbelt region. There is no apparent grazing across the area. When the fire experiment was established, the site was nested within a broader area of old-growth vegetation. Over the last 20-plus years, the wider area has been transformed progressively via wood harvesting for charcoal and land acquisitions by smallholders. Unfortunately, the Mwekera fire experiment is unreplicated, although its importance to understanding the ecology of the region cannot be overstated, because other regional fire experiments, e.g. in Ndola (Trapnell, 1959), have progressively been abandoned, and with none compiling detailed floristic inventories of the ground layer.

The experiment consists of three sites of 0.4 ha, each representing one fire treatment (Chidumayo, 1997). Two sites are burned annually in the late (September–October) and early (May–June) dry season, and one is unburned (Fig. 1B–D). Annual burning is commonly observed across African savannas of intermediate rainfall (~1000 mm year^−1^) (Lehmann *et al.*, 2011). Currently, the three sites of late-, early- and no-fire treatments have 15, 75 and 80 % tree cover, respectively, measured in 2022. The treatments are adjacent and divided by fire breaks (and buffer zones) 5–10 m in width as cleared paths. In 1994, an accidental intense fire affected all three treatments (Chidumayo, 1997), and thus the no-fire treatment was undisturbed for 25 years before our data collection, while the fire treatments remain consistently imposed on the late- and early-fire sites.

### Data collection and preparation

Ground layer species composition was sampled using the Global Grassy Group protocol (Lehmann *et al.*, 2022). In each experimental site, two perpendicularly crossing transects were set up. On each half-transect of 25 m in length, five circular plots of 1 m in diameter were located 5 m apart, in addition to one plot on the cross-section. Consequently, 21 permanently marked plots were sampled at each site, totalling an area of ~16.5 m^2^. Measurements were repeated in the same plots five times at 6-week intervals over the wet season to the early dry season (January, February, April, May and June) in 2020, with the expectation that repeated sampling would provide a full picture of the species composition present. We note a potential margin of error in the exact position of some plots during resampling because of the complexity of data collection over the pandemic. The early dry-season fire took place after the May sampling. Within each plot, all ground layer species (except for tree seedlings) were recorded as presence–absence data. We attached labels with voucher identities to encountered species to identify them at the time of flowering. Vouchers were collected for most species and later identified to confirm field-assigned names and lodged with the Royal Botanic Garden Edinburgh and the National Herbarium in Kitwe. Tree species present in the three sites were identified in June–August 2021. Species names were cleaned and standardized using Plants of the World Online (POWO, 2023).

### Plant functional groups

All ground layer species were recorded and, at a minimum, assigned to one of seven groups: C_4_ grass, C_3_ grass, sedge, non-graminoid monocot, dicot, geoxyle and fern. These groups were assigned on the basis that species composing these groups might have different responses to fire, light availability and microclimates. The chosen categories are a mix of functional and taxonomic groupings, but we refer to them below simply as ‘functional groups’ for clarity.

Grasses (Poaceae) were checked for photosynthetic pathway (C_4_/C_3_) based on Sage (2016). We expected highest C_4_ grass richness in the late-fire treatment owing to fire intensity promoting homogeneity of fuel consumption supporting available niche space for C_4_ grass colonization and persistence. Fewer C_4_ grass species were expected in the early-fire treatment where, after decades, fire is patchy, leading to a partial litter component in the ground layer. It was expected that in the no-fire treatment, with high tree cover, low ground layer light availability and moist microclimate, C_4_ grasses would not be supported. C_3_ grasses can be common in shaded sites and were expected to be found in the no-fire treatment, particularly *Oplismenus hirtellus*. Sedges (Cyperaceae) can occupy diverse environments, but in tropical environments are most often associated with open, wet conditions (Browning *et al.*, 2020), although there is a limited understanding of savanna sedge ecology because they have been little studied (for an exception, see Medwecka-Kornaś and Kornaś, 1985). Non-graminoid monocots (sometimes positioned within forbs) are represented by diverse families, including Orchidaceae, Costaceae, Liliaceae, Iridaceae and Commelinaceae, and are highly species rich. Many grow from underground storage structures, such as bulbs and fleshy rhizomes. This is a functionally diverse group, with some species resilient to disturbance and others recruiting in shaded environments. Thus, we expected species turnover across treatments but with some shared species across treatments. Dicots are the most diverse component of a savanna flora but are poorly studied. Common families include the Asteraceae, Fabaceae and Apocynaceae. Within this broad group, some species could be distinguished as annual or perennial, herbaceous or shrubby, although habit can be highly variable, and for this analysis we were unable to specify life history further with confidence. Among dicots, we expected species unique to each fire treatment. Future improved functional differentiation of dicots will be developed when species can be categorized using a combination of above-ground and belowground traits related to recruitment and resilience in open vs. closed environments. As a category, dicot has been used in previous studies (Kauffman *et al.*, 1994; Cleary and Eichhorn, 2018), whereas others have positioned dicots within forbs (Wragg *et al.*, 2018). We separated geoxyles, as defined by Pausas *et al.* (2018), from the general category of dicots, considering their unique ecology (Meller *et al.*, 2022) and likely long-lived nature. This group included underground trees and with the expectation of geoxyles being strongly associated with fire.

Separately, all trees of ≥2 cm in diameter at breast height present at the three experimental sites were recorded and identified to species. At a local scale, fire can act as a filtering mechanism that removes fire-sensitive species, and this has been well demonstrated in the tree flora of the site and the region. Chidumayo (1997) found that tree richness in the early-fire treatment was higher than in the late- and no-fire treatments, with some species unique to each treatment. However, the lower richness in the no-fire treatment was suggested to be the result of an accidental fire. We did not expect a substantial compositional change across the treatments since the mid-1990s.

### Analyses

The analyses presented operate at two scales. These are: (1) the site/treatment level, which are analyses of species composition across the fire treatments for the ground layer and tree floras; and (2) the plot level, which are analyses of the ground layer species richness patterns, within 1-m-diameter circular plots measured over five repeat surveys throughout January to June 2020 in each treatment. Data were analysed using R v.4.2.1 (R Core Team, 2022) and the following R packages: betapart v.1.6 (Baselga *et al.*, 2023), cowplot v.1.1.1 (Wilke, 2020), eulerr v.7.0.0 (Larsson and Gustafsson, 2018; Larsson, 2022), ggeffects v.1.3.1 (Lüdecke, 2018), gridExtra v.2.3 (Auguie, 2017), iNEXT v.3.0.0 (Chao *et al.*, 2014; Hsieh *et al.*, 2022), lme4 v.1.1.34 (Bates *et al.*, 2015), performance v.0.10.4 (Lüdecke *et al.*, 2021), sjPlot v.2.8.15 (Lüdecke, 2023) and tidyverse v.2.0.0 (Wickham *et al.*, 2019). Details on analysis outputs, assumption checks and sensitivity analysis are provided in the Supplementary Data. The data that support the findings of this study, along with R code used for analyses, are available on figshare (Wieczorkowski *et al.*, 2024).

### Site-level species composition

The β-diversity can be used to quantify compositional differentiation between sites and can be assessed with pairwise dissimilarity (Socolar *et al.*, 2016). Pairwise dissimilarity is useful for determining the environmental features (e.g. tree cover, fire regime) that structure β-diversity, because the magnitude of dissimilarity should be correlated with between-site differences in these features (Anderson *et al.*, 2011). We used the β-diversity framework proposed by Baselga (2010) to differentiate β-diversity (β_SOR_; overall β-diversity, measured as Sørensen dissimilarity) into the components of nestedness (β_SNE_) and turnover (β_SIM_). The β_SNE_ is the nestedness component, measured as the nestedness-resultant fraction of Sørensen dissimilarity, and reflects the loss of species (i.e. composition at a site being a subset of a more species-rich site). The β_SIM_ as the turnover component is measured as Simpson dissimilarity and reflects the replacement of species between sites. Species composition across sites was compared visually using area-proportional Euler plots, with division into the seven functional groups described above along with the tree flora. Although both ground layer and tree composition are available at the site level, these measures are not directly comparable owing to the different area-based sampling strategies required. All tree stems of ≥2 cm diameter at breast height were identified within the sites, whereas ground layer plant species composition has been obtained from 21 plots per site.

### Plot-level species richness

To quantify the effect of fire treatment, total plot-level richness and the plot-level richness of the seven plant functional groups were assessed using generalized linear models (GLMs) with a Poisson distribution. The analyses included one fixed effect of fire treatment and a random effect of the month to account for the variability introduced by the month of sampling. Models were interpreted using the frequentist statistical framework and a significance level of 0.05. The Supplementary Data contains histograms of residuals (Supplementary Data Fig. S1), results of overdispersion tests (Supplementary Data Table S1) and model output summaries (Supplementary Data Table S2). We also ran a sensitivity test, in which we incorporated a random effect of plot, considering that species richness could be more similar across different months where the same plot was resampled (Supplementary Data Fig. S2).

### Sampling completeness

To understand ground layer sampling completeness, we extrapolated species richness to a sample size of 51 plots (~40 m^2^) for total richness (apart from grasses) and separately for grass richness (Supplementary Data Fig. S3), because it often saturates at a lower sample area than other ground layer plants (Lehmann *et al.*, 2022).

## RESULTS

### Floristic description

In the ground layer, we recorded 140 unique species across the three treatments (Supplementary Data List S1). Of these, 50 were dicots, 26 non-graminoid monocots, 19 C_4_ grasses, 19 geoxyles, 12 sedges, 3 ferns and 3 C_3_ grasses, in addition to 4 grasses with unknown photosynthetic pathway and 4 species of an unclassified functional group. Ground layer was represented by 34 families, with 10 families having five or more species: Poaceae (26), Cyperaceae (12), Fabaceae (12), Asteraceae (11), Rubiaceae (8), Lamiaceae (7), Acanthaceae (6), Commelinaceae (5), Dioscoreaceae (5) and Vitaceae (5). Only two species were non-native to Zambia and/or neighbouring countries: *Acmella radicans* and *Spermacoce ocymoides*. Table 1 provides a summary of floristic diversity in each fire treatment.

**Table 1.** Summary of ground layer floristic diversity in each fire treatment. In the ‘species-rich families’ column, the numbers in parentheses indicate the number of species in a family.

Fire treatment	Frequent species	Species-rich families	Unique families
Late	*Trichanthecium nervatum* (C_3_ grass), *Thunbergia kirkiana* (geoxyle), *Digitaria gazensis* (C_4_ grass)	Poaceae (18), Fabaceae (9), Asteraceae (8)	Euphorbiaceae, Caprifoliaceae, Iridaceae
Early	*Geophila obvallata* subsp. *ioides* (dicot), *Clerodendrum buchneri* (geoxyle), *Dioscorea hirtiflora* (non-graminoid monocot)	Poaceae (13), Fabaceae (8), Cyperaceae (7)	Amaranthaceae, Amaryllidaceae, Begoniaceae, Costaceae, Cucurbitaceae, Liliaceae, Ochnaceae
No	*Geophila obvallata* subsp. *ioides* (dicot), *Dioscorea hirtiflora* (non-graminoid monocot), *Grona adscendens* (dicot)	Poaceae (6), Fabaceae (5)	Passifloraceae

Among trees, 59 species were recorded across the three treatments (Supplementary Data List S2), and 50 were previously recorded by Chidumayo (1997). Fabaceae (24) and Phyllanthaceae (7) were the only families with more than three species. The most common species (based on the number of individuals) in the early-fire treatment were *Brachystegia spiciformis* (28), *Parinari curatellifolia* (26) and *Julbernardia paniculata* (22); in the no-fire treatment *Pseudolachnostylis maprouneifolia* (11), *Baphia bequaertii* (9) and *Uapaca kirkiana* (9); and in the late-fire treatment *P. maprouneifolia* (9), *Combretum* sp. (6) and *U. kirkiana* (6).

### Differences in site-level species composition

In pairwise comparisons of ground layer composition across treatments (Table 2A), late- and no-fire treatments (β_SOR_ = 0.705) were the least similar to each other, with 18 species shared between the two sites. Early- and no-fire treatments were the most similar (β_SOR_ = 0.429), with 40 shared species.

**Table 2.** Treatment β-diversity for ground layer plants (A) and trees (B), with division into β_SIM_ (turnover component, measured as Simpson dissimilarity), β_SNE_ (nestedness component, measured as a nestedness-resultant fraction of Sørensen dissimilarity) and β_SOR_ (overall β-diversity, measured as Sørensen dissimilarity). Ground layer and tree data were collected over 16.5 m^2^ and 0.4 ha (4000 m^2^), respectively, hence they are not directly comparable.

Group	Comparison	β_SIM_	β_SNE_	β_SOR_	Total number of species	Shared number of species
(A) Ground layer plants	Late vs. no	0.647	0.058	0.705	104	18
	Late vs. early	0.592	0.046	0.638	131	29
	Early vs. no	0.216	0.213	0.429	100	40
	All treatments	0.548	0.088	0.636	140	16
(B) Trees	Late vs. no	0.259	0.125	0.385	45	20
	Late vs. early	0.296	0.190	0.486	55	19
	Early vs. no	0.158	0.089	0.247	53	32
	All treatments	0.284	0.152	0.436	59	18

**Fig. 2.**
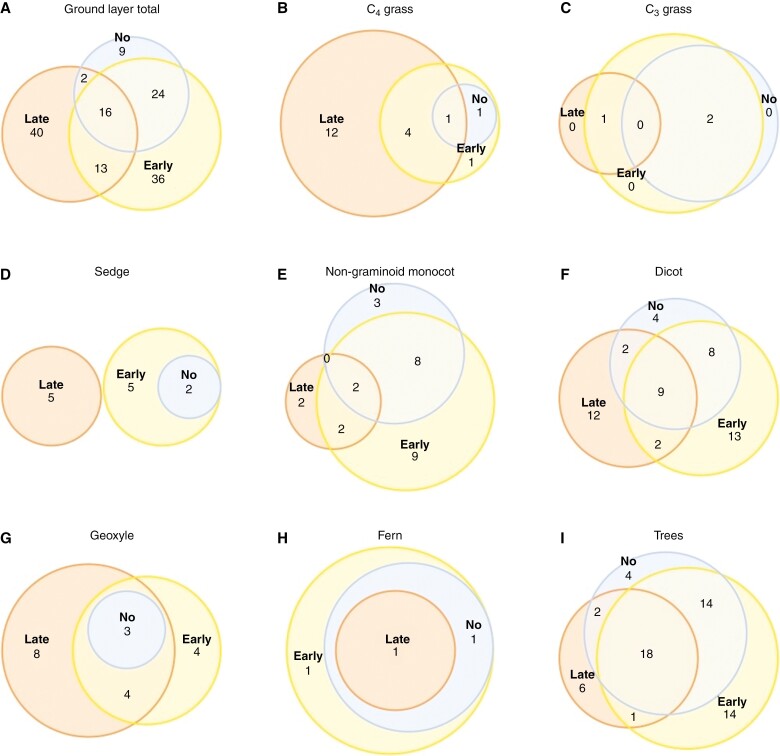
Overview of species composition shared by the three treatments: (A) total ground layer composition; (B–H) with the division to ground layer plant functional groups; and (I) trees.

Communities differed across the three sites, with 89 species recorded in the early-fire treatment. In the late-fire treatment, 71 species were recorded, of which 40 species were unique. The lowest number of species (51) was recorded in the no-fire treatment and 78 % of them were also present in the early-fire treatment, and only nine species were unique (Fig. 2A). There were 16 species that were recorded at least once in each treatment: nine dicots (*Dolichos* sp., *Elephantopus scaber*, *Geophila obvallata* subsp. *ioides*, *Grona adscendens*, *Grona barbata*, *Indigofera livingstoniana*, *Ocimum fimbriatum* var. *fimbriatum*, *Polygala erioptera* and *Sphenostylis stenocarpa*), three geoxyles (*Clerodendrum buchneri*, *Thunbergia kirkiana* and *Triumfetta glechomoides*), two non-graminoid monocots (*Commelina africana* and *Commelina pycnospatha*), one fern (*Nephrolepis undulata*) and one C_4_ grass (*Urochloa brizantha*).

Dicots (Fig. 2F) had similar compositional patterns to the total ground layer (Fig. 2A), whereas other groups showed different patterns. Grasses (Fig. 2B, C) were most numerous in the late-fire treatment, with 12 unique C_4_ species. There was a high turnover in sedge species among treatments, and with no sedge species in common between late-fire and other treatments (Fig. 2D). Late-fire treatment had the fewest non-graminoid monocots (Fig. 2E). Geoxyles were diverse in the fire treatments, with 15 and 11 species recorded in the late- and early-fire treatments, respectively, and three geoxyle species in the no-fire treatment that were found across all treatments (Fig. 2G). Three fern species were recorded (*Nephrolepis undulata*, *Adiantum philippense* subsp. *philippense* and *Adiantum patens* subsp. *oatesii*), with all found in the early-fire treatment (Fig. 2H).

Tree species across the tree treatments (Table 2B; Fig. 2I) were more similar to each other (β_SOR_ = 0.436) than the ground layer species (β_SOR_ = 0.636). Tree composition of the late- and early-fire treatments (β_SOR_ = 0.486) were the least similar to each other, with 19 species shared between the two treatments. Early- and no-fire treatments were the most similar (β_SOR_ = 0.247), with 32 shared species. Although the late-fire treatment had the fewest tree species, it had a higher number of unique species than the no-fire treatment.

### Plot-level species richness patterns

**Fig. 3.**
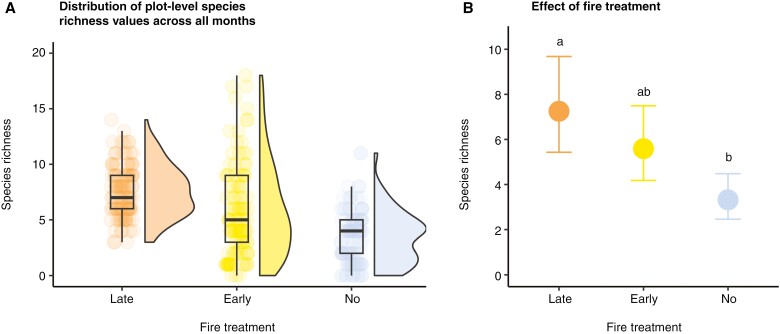
(A) Plot-level species richness in the three treatments. (B) Visualization of the fixed effect of fire treatment. Letters are used to indicate whether 95 % confidence intervals overlap.

The variance in species richness values measured within 1-m-diameter plots in late- and no-fire treatments was low, and in the early-fire treatment it was much more varied, ranging from 0 to a maximum of 18 species per plot in a single month (Table 3; Fig. 3A). Plot-level species richness in the ground layer was significantly higher in late- than in no-fire treatment (Fig. 3B). It was lowest in the no-fire treatment [3.32, 95 % confidence interval (CI): 2.46, 4.48], medium in the early-fire treatment (5.59, 95 % CI: 4.18, 7.49) and highest in the late-fire treatment (7.25, 95 % CI: 5.43, 9.68). A sensitivity test incorporating a random effect of a plot confirmed the same pattern (Supplementary Data Fig. S2).

**Table 3.** Statistical summary for plot-level species richness values of three treatments. A minimum value of zero means that there was only bare ground and/or dead plant litter in the plot.

Fire treatment	Minimum	Maximum	Mean	Median	Interquartile range
Late	3	14	7.59	7	3 (Q1 = 6, Q3 = 9)
Early	0	18	5.86	5	6 (Q1 = 3, Q3 = 9)
No	0	11	3.48	4	3 (Q1 = 2, Q3 = 5)

**Fig. 4.**
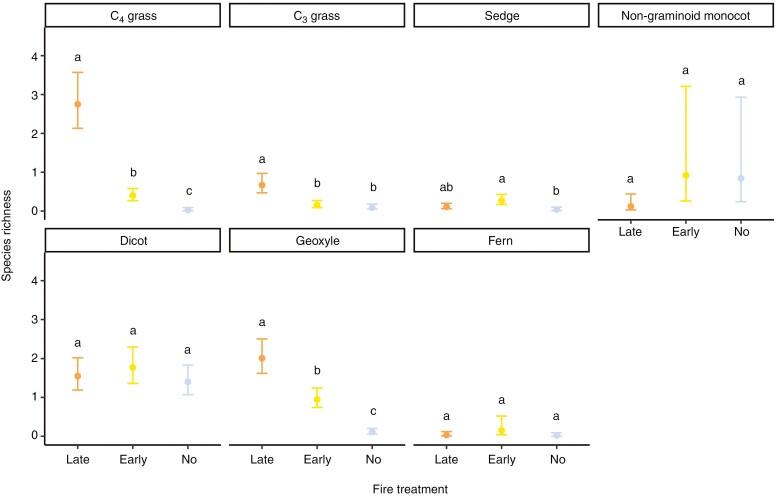
Effect of fire treatment on plot-level species richness of plant functional groups. Letters are used to indicate whether 95 % confidence intervals overlap.

Species richness patterns for individual plant functional groups (Fig. 4) differed from the pattern for the total ground layer species richness (Fig. 3B). Plot species richness of C_4_ grasses differed significantly across the three treatments, with the highest value in late- (2.75, 95 % CI: 2.13, 3.57), medium in early- (0.4, 95 % CI: 0.27, 0.58) and lowest in no-fire treatment (0.03, 95 %CI: 0.01, 0.09). Species richness of geoxyles was the highest in late- (2.01, 95 % CI: 1.62, 2.5), medium in early- (0.95, 95 % CI: 0.74, 1.24) and lowest in no-fire treatment (0.11, 95 % CI: 0.06, 0.2). Among C_3_ grasses, species richness was significantly higher in late-fire treatment (0.67, 95 % CI: 0.47, 0.97) than in the other treatments but did not differ between early- (0.15, 95 % CI: 0.09, 0.27) and no-fire treatments (0.09, 95 % CI: 0.05, 0.18). With respect to sedges, species richness was significantly higher in early- (0.27, 95 % CI: 0.17, 0.43) than in no-fire treatment (0.04, 95 % CI: 0.01, 0.1), and in late-fire treatment (0.11, 95 % CI: 0.06, 0.2) it overlapped with other treatments. Plot-level species richness of dicots, non-graminoid monocots and ferns did not differ between the three treatments.

## DISCUSSION

These are the first data for the wider Miombo region (an area of ~3 × 10^6^ km^2^) to demonstrate that fire has a crucial role in determining ground layer plant diversity and thus in mediating the switch from an open savanna to closed-canopy ecosystem. From the wet to dry season and across the three fire treatments, 140 ground layer species were recorded over ~50 m^2^, with fire-maintained treatments contributing most of the richness and diversity, whereas the no-fire treatment had the lowest total number of species, the lowest number of unique species and the lowest mean species richness per plot.

Fire plays a powerful role in managing plant diversity and thus vegetation structure across the Miombo, where analyses have thus far focused on woody species (e.g. Trapnell, 1959; Ryan and Williams, 2011). Application of three experimental fire treatments led to high compositional turnover among treatments (β_SIM_ = 0.548) and differences in vegetation structure. In the no-fire treatment, there was complete tree canopy closure, with a dense leaf litter layer and almost total absence of grasses. There was a lower but still high canopy cover in the early-fire treatment, with 37 % of the total number of C_4_ grass species recorded present. In the late-fire treatment, there was an open canopy, with 89 % of C_4_ grass species recorded present, of which >40 % were Andropogoneae species that contribute to a high build-up of flammable fuels (Ripley *et al.*, 2015). Long-term fire exclusion has previously been shown to lead to saturation of woody cover and habitat homogenization, equating to the disappearance of light and microclimatic niches necessary for the survival of shade-intolerant species (Pinheiro *et al.*, 2016). The increased woody cover leads to shading that limits ground layer light availability (Pilon *et al.*, 2021), restricting the recruitment and growth of species, whether grasses, geoxyles, dicots or other monocots, reliant on open sunlit environments. Fire exclusion, via an increased number of woody stems, leads to litter accumulation (Pinheiro *et al.*, 2016) that prevents seedling establishment and seed germination of open ecosystem species (Loydi *et al.*, 2013), but conversely, based on our initial field data, we suggest that it supports the germination and seedling survival of regionally widespread tree species, such as of the genera *Brachystegia*, *Julbernardia* and *Isoberlinia*, by reducing drought stress at a key life stage (Chidumayo, 1991). Thus fire-reinforcing feedbacks are likely to operate in two ways, related to: (1) litter and tree species germination; and (2) diverse and abundant C_4_ grass species supporting fire spread, both filtering a fire-sensitive tree flora and creating niche space for an open-adapted ground layer flora. Long-term fire suppression is well recognized as leading to woody encroachment in tropical savannas, causing stark declines in herbaceous plant species richness (Wieczorkowski and Lehmann, 2022) and driving a state transition from savanna to dry forest (Hoffmann *et al.*, 2012), but where implications on plant richness and diversity of a whole ecosystem have been little studied.

Fire and its two seasonal applications in this experiment enabled high species turnover and richness, in contrast to fire exclusion. First, our results on species composition impacts are consistent with similar studies in other regions, indicating high turnover and low nestedness among fire treatments (e.g. Durigan *et al.*, 2020; Gordijn and O’Connor, 2021). Aside from the heterogeneity across treatments, probably coming from the effect of light availability and structural differences changing local microclimates, the compositional differences could also potentially be linked to other factors, such as differences in fire temperatures (Hanley *et al.*, 2003) or post-fire nutrient availability (Hanley and Fenner, 1997). Interestingly, the species composition in the late- and early-fire treatments was more dissimilar (β_SOR_ = 0.638) than the species composition in the the early- and no-fire treatments (β_SOR_ = 0.429). After >60 years, early-fire application does not burn the treatment homogeneously, maintaining a mosaic of litter vs. grass dominance, with a tree component structure similar to the no-fire treatment, while also accommodating a portion of fire-tolerant ground layer flora. Here, early-fire treatment could be considered a site in transition between a savanna and a closed-canopy ecosystem. Half of the β-diversity between early- and no-fire treatments was attributed to nestedness-resultant dissimilarity (β_SNE_ = 0.213), because the no-fire treatment represents a subset of the biota in the richer early-fire treatment. This suggests that no-fire treatment has less favourable environmental conditions (Wright and Reeves, 1992) and does not provide habitat for a unique set of ground layer species, with no clear evidence of refuge or colonization. Second, plot-level species richness in the late-fire treatment was more than twice as high as in no-fire treatment. In the late-fire treatment, there was consistently high species richness in plots [quartile (Q)1 = 6, Q3 = 9], with a continuous grass layer coexisting with a diversity of functional groups. In the early-fire treatment, although the number of species was more variable among plots (Q1 = 3, Q3 = 9), the range in plot-level richness was broadest (0–18) and there was a high diversity of dicots and non-graminoid monocots present in individual plots. In contrast to the no-fire treatment, the application of frequent fire facilitated increased richness, with a diversity of ground layer species benefitting from disturbance and/or open canopy, as observed in savannas worldwide (Peterson and Reich, 2008; Bond and Parr, 2010; Pinheiro *et al.*, 2016). Given that pyrodiversity is not linearly correlated with biodiversity benefits, some fire patterns might be of higher conservation importance (Parr and Andersen, 2006). In the case of the Mwekera experiment, the combination of early and late dry-season fires is crucial to diversifying species composition and increasing species richness.

Plant functional groups did not respond uniformly to the diversity of fire treatments, supported by previous studies (e.g. He *et al.*, 2019; Gordijn and O’Connor, 2021). We observed a qualitative shift in ecosystem dynamics from a C_4_ grass-dominated system in the late-fire treatment to an almost complete absence of C_4_ grasses in the no-fire treatment, with C_4_ grasses unable to persist in shaded conditions (Charles-Dominique *et al.*, 2018; Pilon *et al.*, 2021). Different patterns were observed for C_3_ grasses, with plot-level richness higher in late-fire than in the other treatments owing to the common occurrence of *Trichantecium nervatum* (Paniceae). However, more C_3_ grass species were recorded in other treatments, such as *Oplismenus hirtellus*, a species common in forests and suited to low light levels (Charles-Dominique *et al.*, 2018). Similar to C_4_ grasses, a strong relationship between plot-level species richness and fire treatment was found for geoxyles. Geoxyles regenerate post-fire and are abundant in areas of frequent fire, whereas continued fire exclusion leads to the loss of belowground diversity and bud bank size, potentially losing the capacity to recover even when fire is reintroduced (Bombo *et al.*, 2022). Fire can also be the dominant stimulant in the formation and growth of subsurface stems in geoxyles (Chidumayo, 2019). We found that geoxyle richness is facilitated by fire, with species composition in the no-fire treatment being an impoverished subset of late- and early-fire treatments. Consequently, distinguishing the responses of the ground layer with the use of functional groups provided an opportunity for a more precise understanding of biodiversity responses across an environmental gradient.

The ground layer flora of the Miombo region is severely understudied, undersampled and with remarkably little ecological research conducted. Hence, our research provided new insights into plant groups for which there is little ecological research. It was unexpected to find such turnover in sedges. Fire resistance in sedges from Zambia has been demonstrated to be concentrated in Miombo ecosystems and dambo grasslands (Medwecka-Kornaś and Kornaś, 1985). Fire-resistant sedges have adaptations such as buds positioned underground and covered with rhizome scales (e.g. *Cyperus tenuiculmis*, late-fire treatment), formation of a bulb at the base of each year’s culm, which could nearly be considered woody (e.g. *Scleria bulbifera*, late-fire treatment), the storage of considerable water reserves (e.g. *Cyperus angolensis*, late-fire treatment) or flowering soon after fire (Medwecka-Kornaś and Kornaś, 1985), as has been found in Brazilian savannas (Pilon *et al.*, 2023). Adaptations similar to those of sedges were found for ferns, of which many species in the Miombo region (including *Nephrolepis undulata*, all treatments) possess biological and morphological features of advanced pyrophytes (Kornaś, 1978). Many have perennating buds protected by old stipe bases and dense, thick rhizome scales (Kornaś, 1977). Both sedges and ferns displayed nested patterns of species composition, with all species found within early- and/or late-fire treatments, suggesting their common adaptation to fire. The plot-level richness of non-graminoid monocots and dicots was similar across treatments; however, there was still high turnover in species composition between treatments, and future research should aim to understand the life-history strategies and belowground traits of the dicots and non-graminoid monocots that are little studied. For example, numerous Asteraceae species present, such as *Helichrysum kirkii*, although not geoxyles, probably have long, fleshy taproots enabling storage of non-structural carbohydrates and access to water resources. Furthermore, species such as *Costus spectabilis* (Costaceae), well known and widely distributed across Africa, have deep fleshy rhizomes. It is clear from the species composition recorded in both late- and early-fire treatments that much of the biomass of these ecosystems (along with the buds for post-fire regrowth) is located belowground. Recent research demonstrated that the belowground biomass of a geoxylic grassland was almost equal to that of a densely wooded Miombo ecosystem (Gomes *et al.*, 2021), and it would be pertinent to quantify and understand how belowground biomass varies across the three treatments.

Studies of biodiversity impacts of fire that are based on trees alone, where we found tree species composition to be much less impacted by fire than the ground layer, will under-represent the consequences of changing or homogenizing fire regimes. Increasingly, schemes involving fire management are put forward for carbon offsetting and sequestration that are generally based on modelling tree diversity and/or structure (e.g. Tear *et al.*, 2021). Our data demonstrate that widespread fire exclusion or homogenization of fire via the predominant application of early dry-season fire would lead to a contraction of plant diversity and, crucially, the loss of unique biodiversity. Proposed schemes, generally funded through development aid or corporate financing, must account for and balance the needs of biodiversity and the services these ecosystems provide alongside robust quantification of belowground biomass and soil carbon. In the late- and early-fire treatments, we documented a rich, taxonomically and functionally diverse flora that is not only resilient to fire and seasonal drought but that coexists and persists owing to these disturbances. More than two decades ago, the Cerrado was listed as a biodiversity hotspot to recognize its uniqueness and threatened status (Myers *et al.*, 2000). Since then, Brazilian scientists have accelerated their collaboration with and influence on policy to restore fire as a crucial process shaping the flora (Durigan and Ratter, 2016), where also a savanna region perceived as being of less value than the adjacent Amazon suffered twice the rate of land-use conversion (Lehmann and Parr, 2016). A robust understanding of how fire, climate change and land-use conversion will affect the Miombo region requires a holistic consideration of the ecosystems. Therefore, ecological research is now needed to expand the understanding of functional characteristics of the flora of the region, particularly of the diverse ground flora.

Fire should be recognized as a facilitator, and not an inhibitor, of plant species turnover and richness in Miombo ecosystems, with the implication that any homogeneous fire regime can potentially be negative for a rich and unique biodiversity that has not been adequately studied. Our data show the combination of early and late dry-season fires to be crucial locally to diversifying species composition through increasing species turnover and richness by providing a diversity of local-scale environments, which was not found to compromise tree diversity.

## SUPPLEMENTARY DATA

Supplementary data are available at *Annals of Botany* online and consist of the following.

List S1: list of ground layer species. List S2: list of tree species. Table S1: results of the overdispersion test in GLM analyses. Table S2: output summaries of GLM analyses of ground layer richness. Figure S1: histograms of GLM residuals. Figure S2: sensitivity test for the model of total richness. Figure S3: sample-size-based rarefaction/extrapolation curves.

mcae035_suppl_Supplementary_Material
