# Supplementary material for: Fire facilitates ground layer plant diversity in a Miombo ecosystem
Source: Ann Bot. 2024 Mar 4;133(5-6):743–56. doi: 10.1093/aob/mcae035 (PMC11082521; doi:10.1093/aob/mcae035)
Supplement: mcae035_suppl_Supplementary_Material [file mcae035_suppl_supplementary_material.docx]

Supplementary Information

Analysis outputs, assumption checks, and sensitivity analysis

**Fire facilitates ground layer plant diversity in a Miombo ecosystem**

Jakub D. Wieczorkowski*^1,2^, Caroline E.R. Lehmann^1,2,3^, Sally Archibald^3^, Sarah Banda^4^, David J. Goyder^5^, Mokwani Kaluwe^4^, Kondwani Kapinga^6^, Isabel Larridon^5^, Aluoneswi C. Mashau^3,7^, Elina Phiri^4^, Stephen Syampungani^8,9^

^1^ School of GeoSciences, The University of Edinburgh, Edinburgh, EH8 9XP, United Kingdom

^2^ Tropical Diversity, Royal Botanic Garden Edinburgh, Edinburgh, EH3 5LR, United Kingdom

^3^ Centre for African Ecology, School of Animal, Plant and Environmental Sciences, University of the Witwatersrand, Johannesburg, 2050, South Africa

^4^ Herbarium, Division of Forest Research, Forestry Department, P.O. Box 22099, Kitwe, Zambia

^5^ ​​​​Royal Botanic Gardens, Kew, Richmond, Surrey, TW9 3AE, United Kingdom

^6^ Dag Hammarskjöld Institute for Peace and Conflict Studies – Environment, Sustainable Development and Peace, Copperbelt University, P.O. Box 21692, Kitwe, Zambia

^7^ Foundational Research and Services, South African National Biodiversity Institute (SANBI), Private Bag X101, Pretoria, 0184, South Africa

^8^ Oliver R Tambo Africa Research Chair Initiative for Environment and Development, Copperbelt University, P.O. Box 21692, Kitwe, Zambia

^9^ Department of Plant and Soil Sciences, University of Pretoria, Private Bag X20, Hatfield, 0028, Pretoria, South Africa

***Corresponding Author**: [jakub.wieczorkowski@ed.ac.uk](mailto:jakub.wieczorkowski@ed.ac.uk)

# Contents

**List S1.** List of ground layer species 3

**List S2.** List of tree species 8

**Table S1.** Results of the overdispersion test in GLM analyses 11

**Table S2.** Output summaries of GLM analyses of ground layer richness 12

**Figure S1**. Histograms of GLM residuals 14

**Figure S2.** Sensitivity test for the model of total richness 15

**Figure S3.** Sample-size-based rarefaction/extrapolation curves 16

**References**  17

**List S1.** List of ground layer species with family, functional group assignment and information on presence/absence (1/0) in each treatment. 72.1% of species were identified to species level or lower, 17.1% to genus, 7.9% to family, and 2.9% were unidentified. 60% of species were vouchered with 45% of species confirmed to species level or lower, 14.3% to genus, and 0.7% to family.

|  | **Species** | **Family** | **Functional group** | **Late** | **Early** | **No** |
| --- | --- | --- | --- | --- | --- | --- |
| **1** | ***Acalypha allenii*** | **Euphorbiaceae** | **Geoxyle** | **1** | **0** | **0** |
| **2** | ***Acalypha ambigua*** | **Euphorbiaceae** | **Geoxyle** | **1** | **0** | **0** |
| **3** | **Acanthaceae 1** | **Acanthaceae** | **Dicot** | **0** | **1** | **0** |
| **4** | **Acanthaceae 2** | **Acanthaceae** | **Dicot** | **1** | **0** | **1** |
| **5** | ***Achyropsis laniceps*** | **Amaranthaceae** | **Dicot** | **0** | **1** | **0** |
| **6** | ***Acmella radicans*** | **Asteraceae** | **Dicot** | **0** | **1** | **0** |
| **7** | ***Adenia* sp.** | **Passifloraceae** | **Dicot** | **0** | **0** | **1** |
| **8** | ***Adiantum patens* subsp. *oatesii*** | **Pteridaceae** | **Fern** | **0** | **1** | **0** |
| **9** | ***Adiantum philippense* subsp. *philippense*** | **Pteridaceae** | **Fern** | **0** | **1** | **1** |
| **10** | ***Aframomum alboviolaceum*** | **Zingiberaceae** | **Non-graminoid monocot** | **0** | **1** | **1** |
| **11** | ***Agathisanthemum globosum*** | **Rubiaceae** | **Dicot** | **0** | **1** | **1** |
| **12** | ***Amorphophallus abyssinicus* subsp. *unyikae*** | **Araceae** | **Non-graminoid monocot** | **1** | **1** | **0** |
| **13** | ***Andropogon schirensis*** | **Poaceae** | **C4 grass** | **1** | **1** | **0** |
| **14** | ***Anthephora elongata*** | **Poaceae** | **C4 grass** | **1** | **0** | **0** |
| **15** | ***Aristida recta*** | **Poaceae** | **C4 grass** | **1** | **0** | **0** |
| **16** | ***Aspilia natalensis*** | **Asteraceae** | **Geoxyle** | **1** | **1** | **0** |
| **17** | **Asteraceae 1** | **Asteraceae** | **Dicot** | **1** | **1** | **0** |
| **18** | **Asteraceae 2** | **Asteraceae** | **Dicot** | **1** | **0** | **0** |
| **19** | ***Begonia princeae*** | **Begoniaceae** | **Dicot** | **0** | **1** | **0** |
| **20** | ***Bidens steppia*** | **Asteraceae** | **Dicot** | **1** | **0** | **1** |
| **21** | ***Bulbostylis macra*** | **Cyperaceae** | **Sedge** | **1** | **0** | **0** |
| **22** | ***Carex echinochloe*** | **Cyperaceae** | **Sedge** | **0** | **1** | **0** |
| **23** | ***Cephalaria katangensis*** | **Caprifoliaceae** | **Geoxyle** | **1** | **0** | **0** |
| **24** | ***Chamaecrista meelii*** | **Fabaceae** | **Dicot** | **1** | **0** | **0** |
| **25** | ***Chlorophytum* sp.** | **Asparagaceae** | **Non-graminoid monocot** | **0** | **1** | **0** |
| **26** | ***Cissampelos owariensis*** | **Menispermaceae** | **Dicot** | **0** | **1** | **1** |
| **27** | ***Cissus* sp. 1** | **Vitaceae** | **Dicot** | **0** | **0** | **1** |
| **28** | ***Cissus* sp. 2** | **Vitaceae** | **Dicot** | **0** | **0** | **1** |
| **29** | ***Clematis villosa* subsp. *villosa*** | **Ranunculaceae** | **Geoxyle** | **1** | **1** | **0** |
| **30** | ***Clerodendrum buchneri*** | **Lamiaceae** | **Geoxyle** | **1** | **1** | **1** |
| **31** | ***Clerodendrum* sp.** | **Lamiaceae** | **Dicot** | **0** | **1** | **1** |
| **32** | ***Commelina africana*** | **Commelinaceae** | **Non-graminoid monocot** | **1** | **1** | **1** |
| **33** | ***Commelina pycnospatha*** | **Commelinaceae** | **Non-graminoid monocot** | **1** | **1** | **1** |
| **34** | ***Commelina schweinfurthii* subsp. *ceciliae*** | **Commelinaceae** | **Non-graminoid monocot** | **1** | **1** | **0** |
| **35** | ***Commelina* sp.** | **Commelinaceae** | **Non-graminoid monocot** | **0** | **1** | **0** |
| **36** | ***Costus spectabilis*** | **Costaceae** | **Non-graminoid monocot** | **0** | **1** | **0** |
| **37** | ***Crotalaria calycina*** | **Fabaceae** | **Dicot** | **1** | **0** | **0** |
| **38** | ***Cryptolepis oblongifolia*** | **Apocynaceae** | **Geoxyle** | **1** | **0** | **0** |
| **39** | ***Cryptolepis* sp.** | **Apocynaceae** | **Dicot** | **0** | **1** | **0** |
| **40** | ***Crystallopollen jelfiae*** | **Asteraceae** | **Dicot** | **0** | **1** | **0** |
| **41** | **Cucurbitaceae 1** | **Cucurbitaceae** | **Dicot** | **0** | **1** | **0** |
| **42** | ***Cussonia corbisieri*** | **Araliaceae** | **Geoxyle** | **1** | **1** | **0** |
| **43** | ***Cussonia* sp.** | **Araliaceae** | **Dicot** | **1** | **0** | **0** |
| **44** | ***Cynorkis debilis*** | **Orchidaceae** | **Non-graminoid monocot** | **0** | **1** | **1** |
| **45** | ***Cyperus angolensis*** | **Cyperaceae** | **Sedge** | **1** | **0** | **0** |
| **46** | ***Cyperus cyperoides*** | **Cyperaceae** | **Sedge** | **0** | **1** | **0** |
| **47** | ***Cyperus mapanioides*** | **Cyperaceae** | **Sedge** | **0** | **1** | **0** |
| **48** | ***Cyperus* sp. 1** | **Cyperaceae** | **Sedge** | **1** | **0** | **0** |
| **49** | ***Cyperus* sp. 2** | **Cyperaceae** | **Sedge** | **0** | **1** | **0** |
| **50** | ***Cyperus* sp. 3** | **Cyperaceae** | **Sedge** | **0** | **1** | **1** |
| **51** | ***Cyperus sylvestris*** | **Cyperaceae** | **Sedge** | **0** | **1** | **0** |
| **52** | ***Cyperus tenuiculmis*** | **Cyperaceae** | **Sedge** | **1** | **0** | **0** |
| **53** | ***Cyphostemma* sp. 1** | **Vitaceae** | **Dicot** | **0** | **1** | **0** |
| **54** | ***Cyphostemma* sp. 2** | **Vitaceae** | **Dicot** | **0** | **1** | **0** |
| **55** | ***Cyphostemma vanmeelii*** | **Vitaceae** | **Dicot** | **0** | **1** | **1** |
| **56** | ***Desmodium* sp.** | **Fabaceae** | **Dicot** | **0** | **1** | **0** |
| **57** | ***Digitaria gazensis*** | **Poaceae** | **C4 grass** | **1** | **0** | **0** |
| **58** | ***Diheteropogon amplectens*** | **Poaceae** | **C4 grass** | **1** | **0** | **0** |
| **59** | ***Dioscorea buchananii*** | **Dioscoreaceae** | **Non-graminoid monocot** | **0** | **1** | **0** |
| **60** | ***Dioscorea cochleariapiculata*** | **Dioscoreaceae** | **Non-graminoid monocot** | **0** | **1** | **0** |
| **61** | ***Dioscorea hirtiflora*** | **Dioscoreaceae** | **Non-graminoid monocot** | **0** | **1** | **1** |
| **62** | ***Dioscorea praehensilis*** | **Dioscoreaceae** | **Non-graminoid monocot** | **0** | **1** | **1** |
| **63** | ***Dolichos* sp.** | **Fabaceae** | **Dicot** | **1** | **1** | **1** |
| **64** | ***Dracaena* sp.** | **Asparagaceae** | **Non-graminoid monocot** | **0** | **0** | **1** |
| **65** | ***Elephantopus scaber*** | **Asteraceae** | **Dicot** | **1** | **1** | **1** |
| **66** | ***Eragrostis racemosa*** | **Poaceae** | **C4 grass** | **1** | **0** | **0** |
| **67** | ***Eulophia* sp.** | **Orchidaceae** | **Non-graminoid monocot** | **0** | **0** | **1** |
| **68** | **Fabaceae 1** | **Fabaceae** | **Dicot** | **0** | **1** | **0** |
| **69** | ***Fadogia cienkowskii* var. *cienkowskii*** | **Rubiaceae** | **Geoxyle** | **1** | **0** | **0** |
| **70** | ***Fadogia triphylla*** | **Rubiaceae** | **Geoxyle** | **1** | **0** | **0** |
| **71** | ***Geophila obvallata* subsp*. ioides*** | **Rubiaceae** | **Dicot** | **1** | **1** | **1** |
| **72** | ***Gladiolus gregarius*** | **Iridaceae** | **Non-graminoid monocot** | **1** | **0** | **0** |
| **73** | ***Gloriosa lindenii*** | **Colchicaceae** | **Non-graminoid monocot** | **0** | **1** | **1** |
| **74** | ***Grona adscendens*** | **Fabaceae** | **Dicot** | **1** | **1** | **1** |
| **75** | ***Grona barbata*** | **Fabaceae** | **Dicot** | **1** | **1** | **1** |
| **76** | ***Haumaniastrum villosum*** | **Lamiaceae** | **Dicot** | **1** | **0** | **0** |
| **77** | ***Helichrysum kirkii* var. *petersii*** | **Asteraceae** | **Dicot** | **1** | **0** | **0** |
| **78** | ***Heteropholis* sp.** | **Poaceae** | **C4 grass** | **0** | **1** | **0** |
| **79** | ***Heteropholis sulcata*** | **Poaceae** | **C4 grass** | **1** | **0** | **0** |
| **80** | ***Hyparrhenia bracteata*** | **Poaceae** | **C4 grass** | **1** | **0** | **0** |
| **81** | ***Hyparrhenia filipendula*** | **Poaceae** | **C4 grass** | **1** | **1** | **0** |
| **82** | ***Hyparrhenia newtonii* var. *newtonii*** | **Poaceae** | **C4 grass** | **1** | **0** | **0** |
| **83** | ***Hyparrhenia welwitschii*** | **Poaceae** | **C4 grass** | **1** | **1** | **0** |
| **84** | ***Hypericophyllum angolense*** | **Asteraceae** | **Dicot** | **1** | **0** | **0** |
| **85** | ***Hypoestes forskaolii*** | **Acanthaceae** | **Geoxyle** | **1** | **1** | **0** |
| **86** | ***Indigofera livingstoniana*** | **Fabaceae** | **Dicot** | **1** | **1** | **1** |
| **87** | ***Indigofera sutherlandioides*** | **Fabaceae** | **Geoxyle** | **0** | **1** | **0** |
| **88** | ***Justicia elegantula*** | **Acanthaceae** | **Geoxyle** | **0** | **1** | **0** |
| **89** | ***Lactuca setosa*** | **Asteraceae** | **Dicot** | **0** | **1** | **0** |
| **90** | ***Lilium* sp. 1** | **Liliaceae** | **Non-graminoid monocot** | **0** | **1** | **0** |
| **91** | ***Lilium* sp. 2** | **Liliaceae** | **Non-graminoid monocot** | **0** | **1** | **0** |
| **92** | ***Lilium* sp. 3** | **Liliaceae** | **Non-graminoid monocot** | **0** | **1** | **0** |
| **93** | ***Malaxis katangensis*** | **Orchidaceae** | **Non-graminoid monocot** | **0** | **1** | **1** |
| **94** | ***Microchloa caffra*** | **Poaceae** | **C4 grass** | **1** | **0** | **0** |
| **95** | ***Murdannia simplex*** | **Commelinaceae** | **Non-graminoid monocot** | **1** | **0** | **0** |
| **96** | ***Nephrolepis undulata*** | **Polypodiaceae** | **Fern** | **1** | **1** | **1** |
| **97** | ***Nervilia kotschyi* var. *kotschyi*** | **Orchidaceae** | **Non-graminoid monocot** | **0** | **1** | **1** |
| **98** | ***Ochna pygmaea*** | **Ochnaceae** | **Geoxyle** | **0** | **1** | **0** |
| **99** | ***Ocimum fimbriatum* var. *fimbriatum*** | **Lamiaceae** | **Dicot** | **1** | **1** | **1** |
| **100** | ***Oplismenus hirtellus*** | **Poaceae** | **C3 grass** | **0** | **1** | **1** |
| **101** | ***Orthosiphon allenii*** | **Lamiaceae** | **Geoxyle** | **0** | **1** | **0** |
| **102** | ***Oxytenanthera abyssinica*** | **Poaceae** | **C3 grass** | **0** | **1** | **1** |
| **103** | ***Plectranthus* sp.** | **Lamiaceae** | **Dicot** | **0** | **1** | **1** |
| **104** | ***Poaceae* 1** | **Poaceae** | **NA** | **0** | **1** | **0** |
| **105** | ***Poaceae* 2** | **Poaceae** | **NA** | **0** | **1** | **1** |
| **106** | ***Poaceae* 3** | **Poaceae** | **NA** | **0** | **1** | **0** |
| **107** | ***Poaceae* 4** | **Poaceae** | **NA** | **0** | **0** | **1** |
| **108** | ***Polygala erioptera*** | **Polygalaceae** | **Dicot** | **1** | **1** | **1** |
| **109** | ***Rhynchosia* sp.** | **Fabaceae** | **Dicot** | **1** | **0** | **0** |
| **110** | ***Rottboellia cochinchinensis*** | **Poaceae** | **C4 grass** | **0** | **1** | **1** |
| **111** | **Rubiaceae 1** | **Rubiaceae** | **Dicot** | **0** | **1** | **0** |
| **112** | ***Scadoxus multiflorus*** | **Amaryllidaceae** | **Non-graminoid monocot** | **0** | **1** | **0** |
| **113** | ***Schizachyrium brevifolium*** | **Poaceae** | **C4 grass** | **1** | **0** | **0** |
| **114** | ***Scleria bulbifera*** | **Cyperaceae** | **Sedge** | **1** | **0** | **0** |
| **115** | ***Scleria* sp.** | **Cyperaceae** | **Sedge** | **0** | **1** | **1** |
| **116** | ***Scutellaria schweinfurthii subsp. paucifolia*** | **Lamiaceae** | **Geoxyle** | **1** | **0** | **0** |
| **117** | ***Sida urens*** | **Malvaceae** | **Dicot** | **0** | **1** | **1** |
| **118** | ***Spermacoce chaetocephala*** | **Rubiaceae** | **Dicot** | **1** | **1** | **0** |
| **119** | ***Spermacoce dibrachiata*** | **Rubiaceae** | **Dicot** | **1** | **0** | **0** |
| **120** | ***Spermacoce ocymoides*** | **Rubiaceae** | **Dicot** | **1** | **0** | **0** |
| **121** | ***Sphenostylis stenocarpa*** | **Fabaceae** | **Dicot** | **1** | **1** | **1** |
| **122** | ***Stephania abyssinica*** | **Menispermaceae** | **Dicot** | **0** | **1** | **1** |
| **123** | ***Stylochaeton puberulum*** | **Araceae** | **Non-graminoid monocot** | **0** | **0** | **1** |
| **124** | ***Tacca leontopetaloides*** | **Dioscoreaceae** | **Non-graminoid monocot** | **0** | **1** | **1** |
| **125** | ***Thunbergia kirkiana*** | **Acanthaceae** | **Geoxyle** | **1** | **1** | **1** |
| **126** | ***Thunbergia lancifolia*** | **Acanthaceae** | **Geoxyle** | **1** | **0** | **0** |
| **127** | ***Thyrsia huillensis*** | **Poaceae** | **C4 grass** | **1** | **0** | **0** |
| **128** | ***Trichanthecium nervatum*** | **Poaceae** | **C3 grass** | **1** | **1** | **0** |
| **129** | ***Tristachya superba*** | **Poaceae** | **C4 grass** | **1** | **0** | **0** |
| **130** | ***Triumfetta angolensis*** | **Malvaceae** | **Dicot** | **0** | **1** | **1** |
| **131** | ***Triumfetta glechomoides*** | **Malvaceae** | **Geoxyle** | **1** | **1** | **1** |
| **132** | ***Triumfetta setulosa*** | **Malvaceae** | **Dicot** | **0** | **0** | **1** |
| **133** | ***Unknown* 1** | **Unknown** | **Unknown** | **0** | **1** | **0** |
| **134** | ***Unknown* 2** | **Unknown** | **Unknown** | **1** | **0** | **0** |
| **135** | ***Unknown* 3** | **Unknown** | **Unknown** | **0** | **1** | **1** |
| **136** | ***Unknown* 4** | **Unknown** | **Unknown** | **0** | **0** | **1** |
| **137** | ***Urochloa brizantha*** | **Poaceae** | **C4 grass** | **1** | **1** | **1** |
| **138** | ***Urochloa platynota*** | **Poaceae** | **C4 grass** | **1** | **1** | **0** |
| **139** | ***Vernoniastrum latifolium*** | **Asteraceae** | **Dicot** | **1** | **0** | **0** |
| **140** | ***Zornia glochidiata*** | **Fabaceae** | **Dicot** | **1** | **0** | **0** |

**List S2.** List of tree species with family and information on presence/absence (1/0) in each treatment.

|  | **Species** | **Family** | **Late** | **Early** | **No** |
| --- | --- | --- | --- | --- | --- |
| **1** | ***Afzelia quanzensis*** | **Fabaceae** | **1** | **0** | **1** |
| **2** | ***Albizia adianthifolia*** | **Fabaceae** | **1** | **1** | **1** |
| **3** | ***Albizia amara*** | **Fabaceae** | **0** | **1** | **0** |
| **4** | ***Albizia antunesiana*** | **Fabaceae** | **1** | **1** | **1** |
| **5** | ***Albizia versicolor*** | **Fabaceae** | **0** | **1** | **1** |
| **6** | ***Anisophyllea boehmii*** | **Anisophylleaceae** | **1** | **1** | **1** |
| **7** | ***Annona senegalensis*** | **Annonaceae** | **0** | **1** | **1** |
| **8** | ***Baphia bequaertii*** | **Fabaceae** | **1** | **1** | **1** |
| **9** | ***Bobgunnia madagascariensis*** | **Fabaceae** | **0** | **0** | **1** |
| **10** | ***Brachystegia boehmii*** | **Fabaceae** | **1** | **1** | **1** |
| **11** | ***Brachystegia floribunda*** | **Fabaceae** | **1** | **1** | **1** |
| **12** | ***Brachystegia longifolia*** | **Fabaceae** | **0** | **1** | **1** |
| **13** | ***Brachystegia manga*** | **Fabaceae** | **0** | **1** | **0** |
| **14** | ***Brachystegia* sp.** | **Fabaceae** | **0** | **1** | **0** |
| **15** | ***Brachystegia spiciformis*** | **Fabaceae** | **1** | **1** | **1** |
| **16** | ***Brachystegia utilis*** | **Fabaceae** | **0** | **1** | **1** |
| **17** | ***Bridelia micrantha*** | **Phyllanthaceae** | **0** | **1** | **0** |
| **18** | ***Canthium* sp.** | **Rubiaceae** | **0** | **1** | **0** |
| **19** | ***Combretum pisoniiflorum*** | **Combretaceae** | **0** | **1** | **0** |
| **20** | ***Combretum* sp.** | **Combretaceae** | **1** | **1** | **1** |
| **21** | ***Combretum zeyheri*** | **Combretaceae** | **0** | **1** | **0** |
| **22** | ***Dalbergia lanceolaria* subsp. *paniculata*** | **Fabaceae** | **0** | **1** | **0** |
| **23** | ***Dalbergiella nyasae*** | **Fabaceae** | **0** | **1** | **0** |
| **24** | ***Diospyros batocana*** | **Ebenaceae** | **0** | **1** | **0** |
| **25** | ***Erythrina abyssinica*** | **Fabaceae** | **0** | **1** | **0** |
| **26** | ***Erythrophleum africanum*** | **Fabaceae** | **1** | **1** | **1** |
| **27** | ***Flacourtia indica*** | **Salicaceae** | **0** | **1** | **1** |
| **28** | ***Gardenia imperialis*** | **Rubiaceae** | **0** | **1** | **0** |
| **29** | ***Hexalobus monopetalus*** | **Annonaceae** | **0** | **1** | **1** |
| **30** | ***Hymenocardia acida*** | **Phyllanthaceae** | **1** | **0** | **0** |
| **31** | ***Isoberlinia angolensis*** | **Fabaceae** | **0** | **0** | **1** |
| **32** | ***Julbernardia paniculata*** | **Fabaceae** | **1** | **1** | **1** |
| **33** | ***Landolphia kirkii*** | **Apocynaceae** | **1** | **0** | **0** |
| **34** | ***Lannea discolor*** | **Anacardiaceae** | **0** | **0** | **1** |
| **35** | ***Lannea schweinfurthii* var. *stuhlmannii*** | **Anacardiaceae** | **0** | **1** | **1** |
| **36** | ***Magnistipula butayei*** | **Chrysobalanaceae** | **0** | **1** | **1** |
| **37** | ***Markhamia obtusifolia*** | **Bignoniaceae** | **1** | **0** | **0** |
| **38** | ***Marquesia macroura*** | **Dipterocarpaceae** | **0** | **1** | **1** |
| **39** | ***Monotes africanus*** | **Dipterocarpaceae** | **1** | **1** | **1** |
| **40** | ***Monotes* sp.** | **Dipterocarpaceae** | **1** | **0** | **0** |
| **41** | ***Ochna pulchra*** | **Ochnaceae** | **1** | **1** | **1** |
| **42** | ***Parinari curatellifolia*** | **Chrysobalanaceae** | **1** | **1** | **1** |
| **43** | ***Pericopsis angolensis*** | **Fabaceae** | **1** | **1** | **1** |
| **44** | ***Phyllanthus muellerianus*** | **Phyllanthaceae** | **0** | **1** | **1** |
| **45** | ***Piliostigma thonningii*** | **Fabaceae** | **0** | **1** | **0** |
| **46** | ***Pseudolachnostylis maprouneifolia*** | **Phyllanthaceae** | **1** | **1** | **1** |
| **47** | ***Pterocarpus angolensis*** | **Fabaceae** | **1** | **1** | **1** |
| **48** | ***Rothmannia engleriana*** | **Rubiaceae** | **1** | **1** | **0** |
| **49** | ***Searsia longipes*** | **Anacardiaceae** | **0** | **0** | **1** |
| **50** | ***Strychnos cocculoides*** | **Loganiaceae** | **1** | **0** | **0** |
| **51** | ***Strychnos innocua*** | **Loganiaceae** | **0** | **1** | **1** |
| **52** | ***Syzygium coarctatum*** | **Myrtaceae** | **1** | **1** | **1** |
| **53** | ***Syzygium guineense*** | **Myrtaceae** | **0** | **1** | **1** |
| **54** | ***Uapaca kirkiana*** | **Phyllanthaceae** | **1** | **1** | **1** |
| **55** | ***Uapaca nitida*** | **Phyllanthaceae** | **0** | **1** | **1** |
| **56** | ***Uapaca sansibarica*** | **Phyllanthaceae** | **0** | **1** | **1** |
| **57** | ***Vachellia sieberiana*** | **Fabaceae** | **1** | **0** | **1** |
| **58** | ***Vitex doniana*** | **Lamiaceae** | **1** | **0** | **0** |
| **59** | ***Vitex payos*** | **Lamiaceae** | **0** | **1** | **0** |

**Table S1.** Results of the overdispersion test in GLM analyses. Slight overdispersion has been detected for (a) total and (g) dicot.

|  | Group | Overdispersion test result |
| --- | --- | --- |
| a | Total | 1.34 |
| b | Total (sensitivity test) | 0.72 |
| c | C_4_ grass | 0.72 |
| d | C_3_ grass | 0.64 |
| e | Sedge | 0.96 |
| f | Non-graminoid monocot | 0.73 |
| g | Dicot | 1.13 |
| h | Geoxyle | 0.96 |
| i | Fern | 0.84 |

**Table S2.** Output summaries of GLM analyses of ground layer richness. The table contains Incidence Rate Ratios (IRRs) which should be interpreted as the proportion of the value for the intercept; 95% Confidence Intervals (CI) and the associated p-values (p). Marginal and conditional r-squared values for mixed models are reported which are calculated based on Nakagawa et al. (2017), where Conditional R^2^ takes into account both the variance of fixed and random effects and Marginal R^2^ takes into account only the fixed effects.

|  | **(a) Total** | | |
| --- | --- | --- | --- |
| ***Predictors*** | ***IRR*** | ***CI*** | ***p*** |
| **(Intercept)** | **7.25** | **5.43 – 9.68** | **<0.001** |
| **site [Early]** | **0.77** | **0.69 – 0.86** | **<0.001** |
| **site [No]** | **0.46** | **0.40 – 0.52** | **<0.001** |
| **Random Effects** | | | |
| **σ^2^** | **0.17** | | |
| **τ_00_ _month_** | **0.10** | | |
| **ICC** | **0.37** | | |
| **N _month_** | **5** | | |
| **Observations** | **315** | | |
| **Marginal R^2^ / Conditional R^2^** | **0.280 / 0.550** | | |

|  | **(b) Total (sensitivity test)** | | |
| --- | --- | --- | --- |
| ***Predictors*** | ***IRR*** | ***CI*** | ***p*** |
| **(Intercept)** | **7.13** | **5.18 – 9.81** | **<0.001** |
| **site [Early]** | **0.73** | **0.58 – 0.90** | **0.004** |
| **site [No]** | **0.45** | **0.36 – 0.56** | **<0.001** |
| **Random Effects** | | | |
| **σ^2^** | **0.19** | | |
| **τ_00_ _plotID_** | **0.10** | | |
| **τ_00_ _month_** | **0.10** | | |
| **ICC** | **0.52** | | |
| **N _month_** | **5** | | |
| **N _plotID_** | **63** | | |
| **Observations** | **315** | | |
| **Marginal R^2^ / Conditional R^2^** | **0.223 / 0.625** | | |

**Table S2 (continued)**

|  | **(c) C_4_ grass** | | | **(d) C_3_ grass** | | | **(e) Sedge** | | | **(f) Non-graminoid monocot** | | | **(g) Dicot** | | | **(h) Geoxyle** | | | **(i) Fern** | | |
| --- | --- | --- | --- | --- | --- | --- | --- | --- | --- | --- | --- | --- | --- | --- | --- | --- | --- | --- | --- | --- | --- |
| ***Predictors*** | ***IRR*** | ***CI*** | ***p*** | ***IRR*** | ***CI*** | ***p*** | ***IRR*** | ***CI*** | ***p*** | ***IRR*** | ***CI*** | ***p*** | ***IRR*** | ***CI*** | ***p*** | ***IRR*** | ***CI*** | ***p*** | ***IRR*** | ***CI*** | ***p*** |
| **(Intercept)** | **2.75** | **2.13-3.57** | **<0.001** | **0.67** | **0.47-0.97** | **0.031** | **0.11** | **0.06-0.20** | **<0.001** | **0.12** | **0.03-0.44** | **0.001** | **1.55** | **1.19-2.02** | **0.001** | **2.01** | **1.62-2.50** | **<0.001** | **0.03** | **0.01-0.12** | **<0.001** |
| **site [Early]** | **0.14** | **0.10-0.20** | **<0.001** | **0.23** | **0.14-0.39** | **<0.001** | **2.5** | **1.28-4.87** | **0.007** | **7.74** | **5.02-11.93** | **<0.001** | **1.14** | **0.92-1.40** | **0.224** | **0.47** | **0.37-0.60** | **<0.001** | **5.2** | **2.04-13.22** | **0.001** |
| **site [No]** | **0.01** | **0.00-0.03** | **<0.001** | **0.14** | **0.07-0.26** | **<0.001** | **0.33** | **0.11-1.03** | **0.056** | **7.04** | **4.56-10.89** | **<0.001** | **0.9** | **0.72-1.12** | **0.341** | **0.06** | **0.03-0.10** | **<0.001** | **0.6** | **0.15-2.42** | **0.473** |
| **Random Effects** | | | | | | | | | | | | | | | | | | | | | |
| **σ^2^** | **0.66** | | | **1.45** | | | **2.1** | | | **0.95** | | | **0.49** | | | **0.68** | | | **2.8** | | |
| **τ_00_ _month_** | **0.07** | | | **0.1** | | | **0.08** | | | **1.94** | | | **0.06** | | | **0.04** | | | **1.48** | | |
| **ICC** | **0.09** | | | **0.06** | | | **0.04** | | | **0.67** | | | **0.11** | | | **0.05** | | | **0.34** | | |
| **N_month_** | **5** | | | **5** | | | **5** | | | **5** | | | **5** | | | **5** | | | **5** | | |
| **Observations** | **315** | | | **315** | | | **315** | | | **315** | | | **315** | | | **315** | | | **315** | | |
| **Marginal R^2^ / Conditional R^2^** | **0.829 / 0.846** | | | **0.317 / 0.360** | | | **0.238 / 0.267** | | | **0.235 / 0.748** | | | **0.016 / 0.124** | | | **0.677 / 0.693** | | | **0.166 / 0.454** | | |

**Figure S1**. Histograms of GLM residuals.


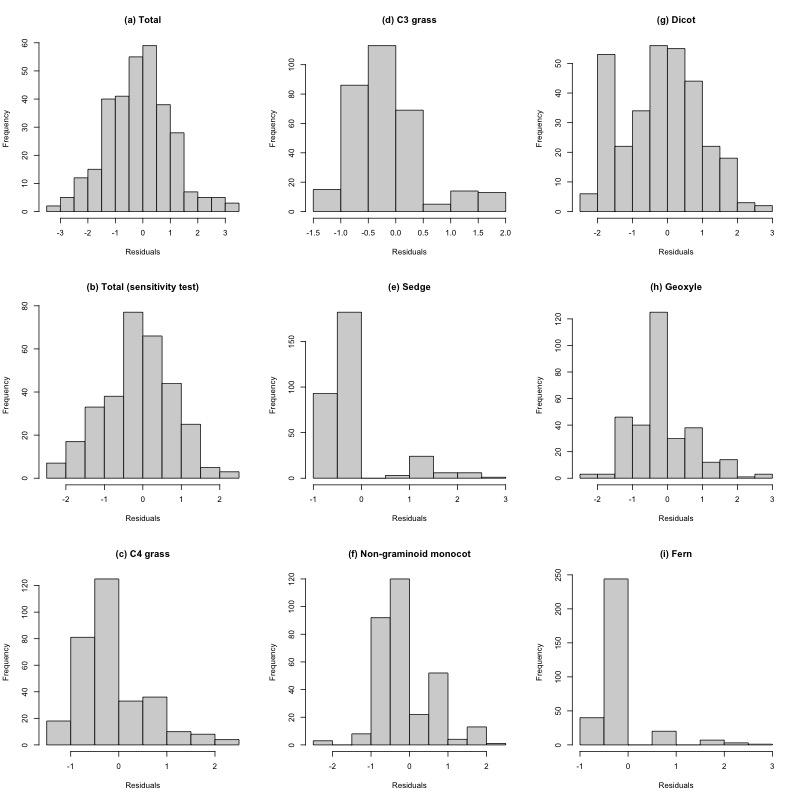


**Figure S2.** Visualisation of the fixed effect of fire treatment in the sensitivity test for the model of total richness which incorporates a random effect of plot. Letters are used to indicate whether 95% Confidence Intervals overlap. The species richness in Late (7.13, 95%CI: 5.18, 9.81) and No fire treatment (3.18, 95%CI: 2.29, 4.41) do not overlap. Species richness in the Early fire treatment (5.18, 95%CI: 3.75, 7.15) overlaps with other treatments.


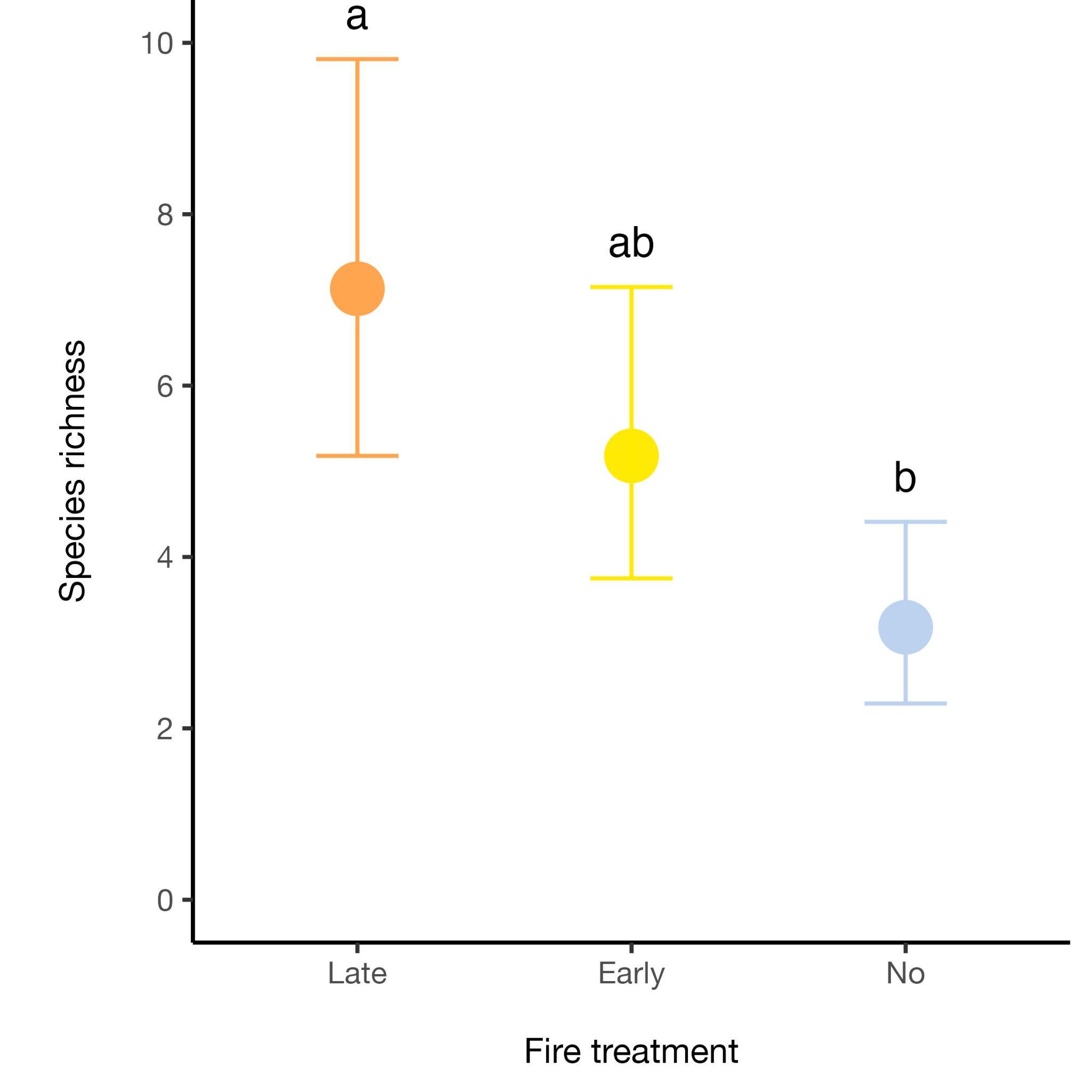


#

**Figure S3.** Sample-size-based rarefaction and extrapolation of species richness at each treatment in each month of sampling. Species richness has been divided into (a) grasses, and (b) all ground layer species except for grasses. The extrapolation was carried out to a sample size of 51 plots (~40 m^2^), using iNEXT (Hsieh et *al.,* 2016) function with an ‘incidence_raw’ data type (species by sampling-units incidence matrix). Grass species richness saturated quicker than non-grasses, however, some species could still have been missed, especially in April and in Late fire treatment. Non-grass plot richness did not saturate in Early and Late fire treatments in any month which means that some species present at the two sites have likely been missed.

Globally, the Brazilian Cerrado is lauded for its plant diversity. Comparing local richness recorded at Mwekera to that of Abreu et al. (2017) across a similarly fire-determined tree cover gradient, mean ground layer species richness was comparable. Extrapolation of Mwekera ground layer richness to an equivalent area as sampled by Abreu et al. (2017) from our April sampling alone would be 34 (No fire), 58 (Early fire), and 60 (Late fire) species compared with their three categories of encroachment of 23, 78 and 81 species (over 40 m^2^, excluding trees and palms).

#
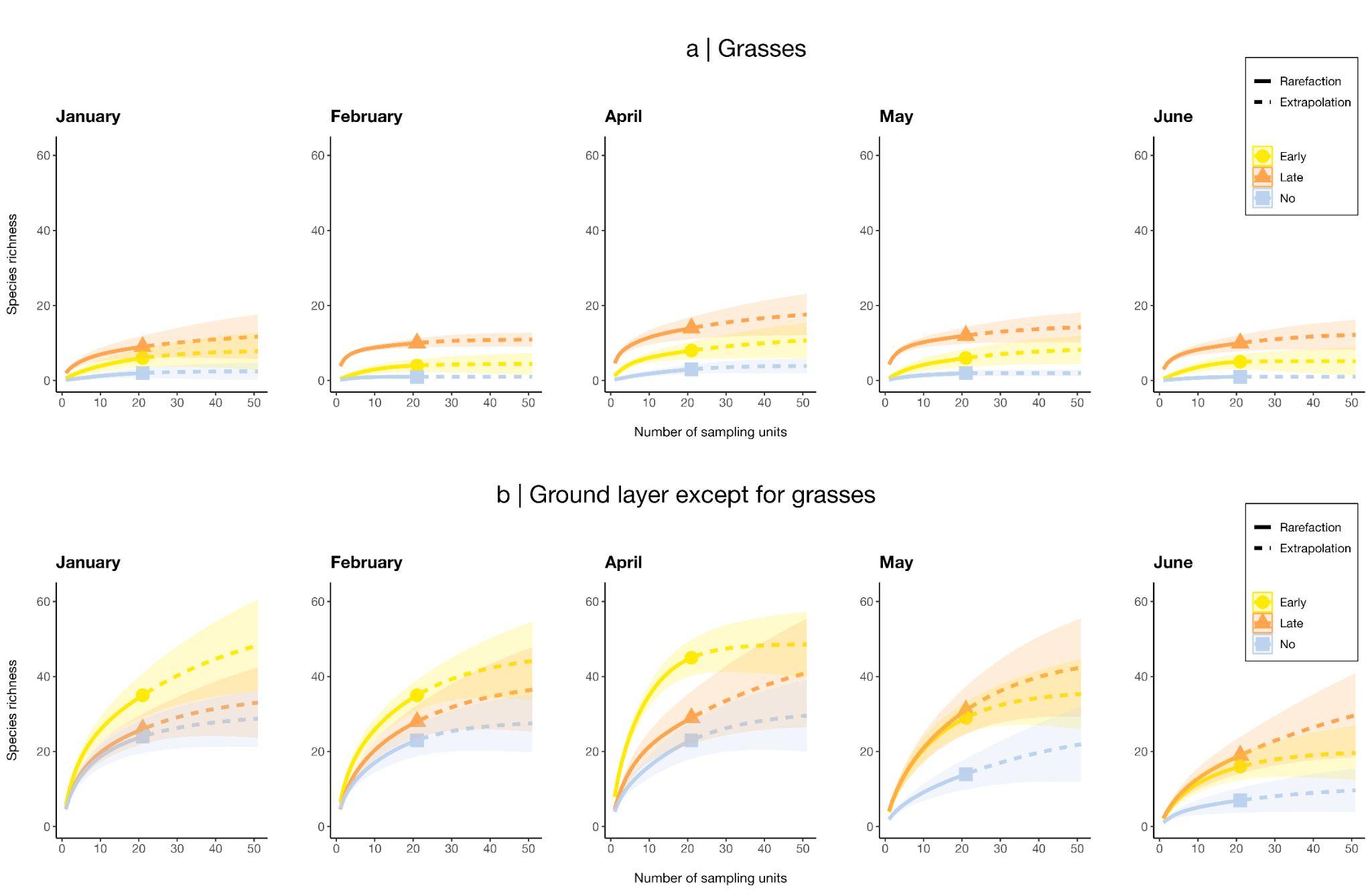


# References

Abreu RCR, Hoffmann WA, Vasconcelos HL, Pilon NA, Rossatto DR, Durigan G. 2017. The biodiversity cost of carbon sequestration in tropical savanna. *Science Advances* 3: e1701284.

Hsieh TC, Ma KH, Chao A. 2016. iNEXT: an R package for rarefaction and extrapolation of species diversity (Hill numbers). *Methods in Ecology and Evolution* 7: 1451–1456.

Nakagawa S, Johnson PCD, Schielzeth H. 2017. The coefficient of determination R2 and intra-class correlation coefficient from generalized linear mixed-effects models revisited and expanded. *Journal of The Royal Society Interface* 14: 20170213.
